# *Placental ESRRG-CYP19A1* Expressions and Circulating 17-Beta Estradiol in IUGR Pregnancies

**DOI:** 10.3389/fped.2019.00154

**Published:** 2019-04-24

**Authors:** Gaia Maria Anelli, Chiara Mandò, Teresa Letizia, Martina Ilaria Mazzocco, Chiara Novielli, Fabrizia Lisso, Carlo Personeni, Tarcisio Vago, Irene Cetin

**Affiliations:** ^1^Unit of Obstetrics and Gynecology, Department of Biomedical and Clinical Sciences, ASST Fatebenefratelli Sacco University Hospital, University of Milan, Milan, Italy; ^2^Endocrinology Laboratory, Sacco University Hospital, Milan, Italy; ^3^Unit of Obstetrics and Gynecology, Buzzi University Hospital, Milan, Italy

**Keywords:** intrauterine growth restriction, placenta, 17-β estradiol, estrogen-related receptor γ, cytochrome p450 aromatase

## Abstract

**Introduction:** Sex steroids are regulating factors for intrauterine growth. 17-β Estradiol (E2) is particularly critical to a physiological pregnancy, as increased maternal E2 was correlated to lower fetal weight at delivery. The placenta itself is a primary source of estrogens, synthetized from cholesterol precursors. Cytochrome P450 aromatase (encoded by *CYP19A1* gene) is a rate-limiting enzyme for E2 biosynthesis. *CYP19A1* transcription is supported by Estrogen Related-Receptor Gamma (ERRγ- *ESRRG* gene), which thus has an indirect role in placental steroidogenesis. Here we investigated maternal E2 levels and placental *CYP19A1* and *ESRRG* expressions in pregnancies with IntraUterine Growth Restriction (IUGR).

**Methods:** Singleton pregnancies were studied. E2 was measured in maternal plasma by electrochemiluminescence in 16 term controls and 11 IUGR (classified by umbilical artery doppler pulsatility index) at elective cesarean section, and also in 13 controls during pregnancy at a gestational age comparable to IUGR. *CYP19A1* and *ESRRG* expressions were analyzed in placental tissue. Maternal/fetal characteristics, placental and molecular data were compared among study groups and tested for correlations.

**Results:** Maternal E2 plasma concentrations were significantly decreased in IUGR compared to controls at delivery. When analyzing normal pregnancies at a gestational age similar to IUGR, E2 levels were not different to pathological cases. However, E2 levels at delivery positively correlated with placental efficiency. Placental *CYP19A1* levels were significantly higher in IUGR placental tissue vs. controls, and specifically increased in female IUGR placentas. *ESRRG* expression was not different among groups.

**Discussion:** We report a positive correlation between 17-β Estradiol levels and placental efficiency, that might indicate a disrupted steroidogenesis in IUGR pregnancies. Moreover, we show alterations of *CYP19A1* expression in IUGR placentas, possibly indicating a compensatory effect to the adverse IUGR intrauterine environment, also depending on fetal sex. Further studies are needed to deeper investigate IUGR alterations in the complex interaction among molecules involved in placental steroidogenesis.

## Highlights

- Altered E2 levels suggest disturbances of estrogenic pathways in IUGR pregnancies.
- *ESRRG* and *CYP19A1* alterations may be linked to growth-restriction features.
- *CYP19A1* alteration as a clue of IUGR disrupted placental steroidogenesis.
- Fetal sex may influence *CYP19A1* expression in IUGR placentas.

## Introduction

During pregnancy, a well-functioning placenta is needed to ensure the appropriate growth and development of the fetus (1). Indeed, an “insufficient” placenta is one of the main characteristics of IntraUterine Growth Restriction (IUGR) (2), with decreased oxygen supply and altered nutrient transport to the fetus (3–7). Multiple stimuli occur throughout pregnancy to achieve an appropriate placental vascularization, perfusion and metabolism. Oxygen concentration, energy metabolism, and sex steroids have been described as limiting factors for growth-restriction onset (8–11).

Sex steroids in the fetal and maternal circulations play an important role in determining a physiological placental phenotype (12). Being a developing organ, the placenta undergoes significant anatomical, and functional changes which are partially regulated by steroids, particularly estrogens (13). Indeed, inhibition of estrogen production was reported to result in pregnancy loss in animal models (14, 15). Like other estrogens, 17-β Estradiol (E2) is involved in each pregnancy stage: it promotes endometrium growth, preparing the uterus for embryo's implantation, it regulates uterine vascular adaptations to pregnancy and labor induction (16, 17), and is also critical for trophoblast syncytialization (18). In early pregnancy, high maternal estradiol levels have been reported to correlate with increased risks of low neonatal weight (19), by affecting the expression of *CDKN1C* and *IGF2*, placental imprinted-genes which are relevant to fetal growth (20).

Growing evidence has been reported on the association between altered circulating progesterone and estrogen levels and PreEclampsia (PE) (21–23). Conversely, few progesterone/estrogen profiles have been investigated in pregnancies delivering Small for Gestational Age (SGA) babies. First evidence that maternal 17-β Estradiol concentration may be related to fetal growth restriction derived from a mid-to-late-term pregnancy sheep model (24). Data from human pregnancies were recorded by Salas et al. at 34 gestational weeks. Reduced progesterone and estradiol plasma levels were measured in the maternal plasma of women delivering SGA babies, but no clinical data were reported about intrauterine growth (25).

During pregnancy, the placenta itself becomes the primary source of progesterone (at term, 250 mg/day) and estrogens (at term, 100–120 mg/day), synthetized from both maternal and fetal cholesterol precursors. Indeed, sulfated C-19 androgens, 16α-hydroxylated DHEA-S and DHEA-S are processed into estrogens by the activity of several enzymes involved in steroids biosynthesis (known as steroidogenesis), namely placental STeroid Sulfatase (STS), 3β HydroSteroid Dehydrogenases 1 (HSD1), CYP19 (CYtochrome P450 aromatase), and 17βHSD isoenzymes. Estrogens produced in the placenta are then released into both maternal and fetal circulations (26, 27).

Therefore, several placental genes are involved in steroidogenesis, being critical to sex steroids metabolism. Their expression may thus represent a limiting factor to normal placental development (28).

CYtochrome P450 aromatase, encoded by *CYP19A1*, is a rate-limiting enzyme for sex steroids biosynthesis, particularly for placental 17-β Estradiol. Altered *CYP19A1* gene and protein levels have been recently observed in preeclamptic placentas, associated with a trend to lower estriol and estradiol maternal plasma profiling. Berkane et al. suggested placental hypoxia as a possible causative mechanism for CYP19 dysregulation (29). The consequent downregulation of E2 levels could contribute to the antiangiogenic and vasoconstricted clinical features of PE.

Among the Estrogen Receptors (ER) regulating sex steroids metabolism, Estrogen Related-Receptor (ERR) is becoming a promising topic of interest. ERR is involved in several energetic pathways such as mitochondria replication and Oxidative Phosphorylation (OXPHOS) (30), but is also a critical mediator of multiple metabolic and endocrine signals (31, 32). Its Gamma isoform (*ESRRG*) is predominantly expressed in placenta (33), and regulates trophoblast differentiation. *In vitro* studies have highlighted an oxygen-dependent expression for *ESRRG*, being down-regulated in cytotrophoblast cultures under hypoxia (34, 35).

ESRRG role in regulating placental steroidogenesis (36) has been recently proposed by an *in vitro* model reporting its binding to *CYP19A1* promoter (35), with the consequent induction of Cytochrome p450 aromatase (37). Moreover, silencing of *ESRRG* in extravillous trophoblasts has been recently reported to suppress the expression of HSD17β1, another steroidogenic enzyme critical to the placental conversion of estrone to estradiol (38).

Here we hypothesize alterations of placental steroidogenesis in IUGR compared to normal pregnancies. We measured *ESRRG* and *CYP19A1* gene expression in human IUGR and control pregnancies, in both placental tissue and isolated trophoblast cells, also in relation to IUGR severity. Additionally, we analyzed 17-Beta Estradiol levels in maternal plasma of the same pregnancies to evaluate a potential cause-effect relation with *ESRRG* and *CYP19A1* expression.

## Methods

Pregnant women were enrolled in the Unit of Obstetrics and Gynecology of the “Luigi Sacco Hospital” in Milano. The study protocol was approved by the “L. Sacco Hospital” Ethical Committee and all participants gave their written informed consent.

### Population

Only Caucasian women with singleton spontaneous pregnancies were included in the study. Exclusion criteria were maternal preexisting diseases, alcohol or drug-abuse, maternal and fetal infections, fetal malformations or chromosomal disorders.

Controls (*n* = 17) were term (≥37 weeks) physiological pregnancies with normal intrauterine growth and Appropriate for Gestational Age (AGA) birth weight, according to reference ranges for the Italian population, and delivering by elective cesarean section (CS) [**Controls at Delivery**]; no placental or fetal disease were recorded. Indications for cesarean delivery before labor were breech presentation, repeated cesarean sections, or maternal request.

IntraUterine Growth Restriction **[IUGR]** pregnancies (*n* = 17) were identified *in utero* through longitudinal measurements indicating abdominal circumferences below the 10^th^ percentile of age-related reference values (39) together with a shift from the growth curve >40 centiles (40). IUGR were further evaluated according to umbilical artery Pulsatility Index (PI) (measured by doppler velocimetry) (5, 41) to be classified in two different groups of severity [**Normal PI IUGR** or **Abnormal PI IUGR**].

All IUGR babies were delivered by elective cesarean section.

A further control group (*n* = 13) was enrolled during the III trimester of normal pregnancy (26−32 weeks) to collect maternal plasma samples with a gestational age similar to IUGR, with confirmed AGA birth weight at the time of delivery [**III Trimester Controls**].

### Clinical Data Collection

Medical history, demographic, anthropometric, obstetric, and neonatal data were recorded at recruitment and after delivery. Maternal hemoglobin and hematocrit were measured at 34–36 weeks.

Placental biometry parameters were measured and derived as previously described (42).

### Biological Samples Collection and Analyses

#### 17-Beta Estradiol Analysis in Maternal Plasma

Maternal blood was collected from a radial vein at III trimester (*n* = 13) or at CS for AGA (*n* = 16) and IUGR (*n* = 11) pregnancies. EDTA blood samples were centrifuged at 1500 rpm × 15 min at room temperature and the obtained plasma stored at −80°C. Hemolyzed, icteric, and lipemic plasma samples were excluded.

17-Beta Estradiol concentration was determined using an electrochemiluminescence immunoassay (Estradiol G3 Elecsys Cobas E100, ref. 06656021190) performed on Cobas e411 analyzer, according to the manufacturer's instructions. All plasma samples were diluted 1:10 and analyzed in duplicate; the measurement interval was 549.8–4946 pg/mL. Internal quality controls U1 and U2 (PreciControl Universal, ref. 11731416) were concurrently analyzed with values within the expected ranges. E2 lower limit of detection was 5 pg/mL, while the intra and inter assay coefficients of variation were both ≤10%.

All reagents and instrument were from Roche Diagnostic GmbH, Mannheim, Germany.

#### Tissue Processing, Cells Isolation, RNA Extraction

Placentas were collected and sampled immediately after CS as previously reported (43). After removing the maternal decidua, placental biopsies (~1 cm^3^) were sized from different cotyledons midway between the cord insertion and placental border. Minced placental villi were washed in PBS to be stored at −20°C in RNA Later Solution (Sigma-Aldrich, St. Louis/MO, USA). From tissue fragments preserved in RNA Later (90 mg), RNA was extracted with the RiboPure RNA Purification Kit (Ambion- ThermoFisher Scientific, Austin/TX, USA).

Isolated RNA was then treated with DNase I (New England Biolabs, Ipswich/MA, USA), to remove potentially contaminating DNA; RNA concentrations were measured by NanoDrop ND1000 spectrophotometer (NanoDrop Technologies, Wilmington/DE, USA).

#### *ESRRG* and *CYP19A1* Expression in Placental Tissue

Gene expression of *ESRRG* [Chr.1] and *CYP19A1* [Chr.15] was measured in placental tissue [14 **Controls** and 14 **IUGR**]. For each sample, 1.6 μg of total RNA were reverse-transcribed with High Capacity cDNA Reverse Transcription Kit; cDNA served as template for quantitative Real Time PCR with TaqMan probes for *ESRRG* [assay ID: Hs00976243_m1] and *CYP19A1* [assay ID: Hs00903413_m1]. All samples were reverse-transcribed in duplicate; cDNA was run in triplicate.

Expression levels were calculated using GeNorm method (44) relative to *HPRT* [assay ID: Hs99999909_m1] and *YWHAZ* [assay ID: Hs00237047_m1], selected as placental housekeeping genes (45).

All reagents were supplied by Life Technologies (ThermoFisher Scientific, Foster City/CA, USA).

### Statistical Analyses

Maternal and fetal clinical characteristics and molecular data displayed a normal distribution (Kolmogorov-Smirnov test) and were thus compared among study groups by One-way ANalysis Of VAriance (ANOVA) or independent-samples *t*-test in accordance to the homogeneity of variance (Levene's test).

A Two-way between-groups ANOVA was conducted to assess the impact of IUGR severity and fetal sex (independent variables), as individual or joint effect, on placental genes expression (dependent variables), and applied with the Levene's assumptions. For both ANOVAs, a Tukey's HSD test was run as *post-hoc* test.

Chi-square test was performed to compare fetal sex frequencies among groups, applying Yates continuity correction.

Correlations between clinical/molecular data were assessed using the Pearson product-moment correlation.

Correlations and comparisons between groups were considered statistically significant when *p* < 0.05.

Statistical analyses were performed using SPSS (v.25.00, IBM Statistics, Armonk/NY, USA).

## Results

### Characteristics of the Population

Table 1 presents clinical data in Controls and in IUGR of different severity.

**Table 1:** Maternal, placental, and fetal characteristics in the analyzed population.

	Controls at Delivery[^*^] *n* = 17	**Normal PI IUGR *n* = 8**	**Abnormal PI IUGR *n* = 9**	**III Trimester Controls[^†^] *n* = 13**
**MATERNAL DATA**				
Age, years	34.6 ± 5.5	35.9 ± 4.4^†^	34.0 ± 5.9	30.1 ± 4.0
Pregestational BMI, kg/m^2^	21.3 ± 3.0	21.5 ± 2.2	24.3 ± 4.2	21.4 ± 2.6
Maternal hematocrit, Htc	34.0 ± 1.5	35.7 ± 2.8	34.9 ± 1.7	36.7 ± 5.3
Maternal hemoglobin, Hb	11.3 ± 0.7	11.7 ± 1.1	11.1 ± 1.4	12.4 ± 1.5
**FETAL AND PLACENTAL DATA**				
Gestational age at sampling, wks	39.2 ± 0.4	34.9 ± 3.6	32.6 ± 3.6	32.7 ± 2.5
Gestational age at delivery, wks	39.2 ± 0.4	34.9 ± 3.6^***^^††^	32.6 ± 3.6^***^^†††^	39.6 ± 0.9
Fetal weight, g	3401 ± 330	1768 ± 648^***^^†††^	1501 ± 785^***^^†††^	3419 ± 379
Placental weight, g	457 ± 88	316 ± 129^**^^†^	243 ± 128 ^***^^†††^	471 ± 83
Placenta area, cm^2^	282.9 ± 66.1	189.6 ± 71.6^*^^†^	156.8 ± 64.7^**^^††^	350.9 ± 16.7
Placenta thickness, cm	1.7 ± 0.6	1.5 ± 0.1	1.5 ± 0.4	1.4 ± 0.3

*Data are presented as mean ± standard deviation*.

Post hoc comparisons using the Tukey HSD test are made between IUGR groups vs. Controls at Delivery [*]:

* *p ≤ 0.05*,

** p ≤ 0.01;

*** p ≤ 0.001; or vs. III Trimester Controls [^†^]:

† *p ≤ 0.05*,

†† *p ≤ 0.01*;

††† *p ≤ 0.001*.

*IUGR, Intrauterine Growth Restriction; PI, umbilical artery Pulsatility Index used to define IUGR severity; BMI, Body Mass Index*.

Maternal age and pregestational Body Mass Index (BMI) did not differ among groups.

No remarkable differences in maternal hemoglobin and hematocrit levels were reported.

As expected, gestational age was significantly different among groups [F_(2, 33)_ = 21.7, *p* < 0.001], being lower in growth-restricted babies compared to Controls at Delivery. Fetal weight [F_(2, 33)_ = 43.7, *p* < 0.001], placental weight [F_(2, 31)_ = 11.2, *p* < 0.001], and placental area [F_(2, 25)_ = 9.0, *p* ≤ 0.001] resulted significantly different among groups at delivery, with *post-hoc* test confirming lower fetal-placental parameters for IUGRs vs. term controls. Placental efficiency, calculated at delivery as fetal-placental weight ratio, was significantly decreased in growth-restricted pregnancies [Normal PI IUGR: 5.6 ± 0.6; Abnormal PI IUGR: 5.8 ± 1.0] compared to Controls at Delivery [8.1 ± 2.3] (*Post-Hoc* test: *p* < 0.05 and *p* < 0.01, respectively). No significant differences were found in the distribution of male and female neonates between IUGR and controls [Fetal Sex: χ*2*(1, *n* = 34) = 1.91; *p* = 0.17; phi = 0.30].

Maternal, fetal and placental parameters were similar at delivery (*t*-test: ns) between the two control groups, as shown in Table 1, thus confirming that III Trimester Controls were physiological throughout pregnancy. Gestational age at sampling of III Trimester Controls [32.7 ± 2.5 weeks] was similar to both Normal and Abnormal PI IUGR's [Normal PI: 35.0 ± 3.6; Abnormal PI: 32.6 ± 3.6 weeks] as designed for 17-Beta Estradiol experiments (Table 1).

### 17-Beta Estradiol in Maternal Blood

**Figure 1:**
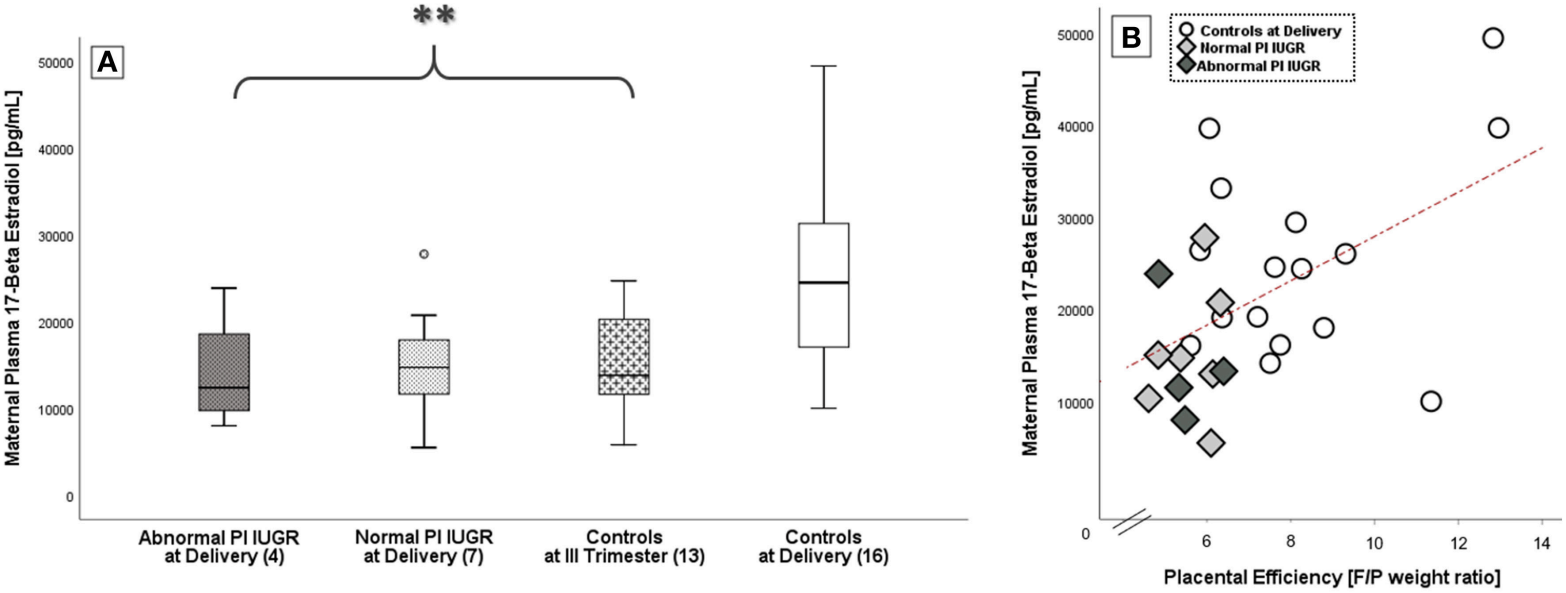
17-Beta estradiol in maternal blood. **(A)** E2 comparison among IUGR with Abnormal and Normal umbilical artery Pulsatility Index- PI, Controls at III Trimester and Controls at Delivery; data shown as Box Plots graphs. Statistical analysis by One-way between-groups ANOVA: ^**^p ≤ 0.01 vs. Controls. **(B)** Significant positive correlation between 17-Beta Estradiol levels and placental efficiency in Controls at Delivery (○), Normal PI (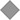), and Abnormal PI (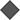) IUGR. Statistical analysis by Pearson product-moment correlation: *r* = 0.51/*p* = 0.01.

Maternal 17-Beta Estradiol levels (E2, pg/mL) were significantly different among groups [F_(3, 39)_ = 4.9, *p* = 0.01] with a large effect size (Eta -η^2^- Squared = 0.50). Plasma E2 had a decreased mean score in both IUGR groups [Normal PI IUGR: 15305.4 ± 7211.1; Abnormal PI IUGR: 14171.3 ± 6828.7] and in III Trimester Controls [14915.0 ± 5877.5] compared to Controls at Delivery [25351.3 ± 10755.4] (*p* = 0.01; Figure 1A). Maternal E2 concentration significantly correlated with gestational age at sampling (*r* = +0.57/*p* < 0.01, data not shown).

Interestingly, E2 levels at delivery were also significantly and positively correlated to placental efficiency (*r* = +0.51/*p* = 0.01; Figure 1B).

### *ESRRG* and *CYP19A1* in Placental Tissue

**Figure 2:**
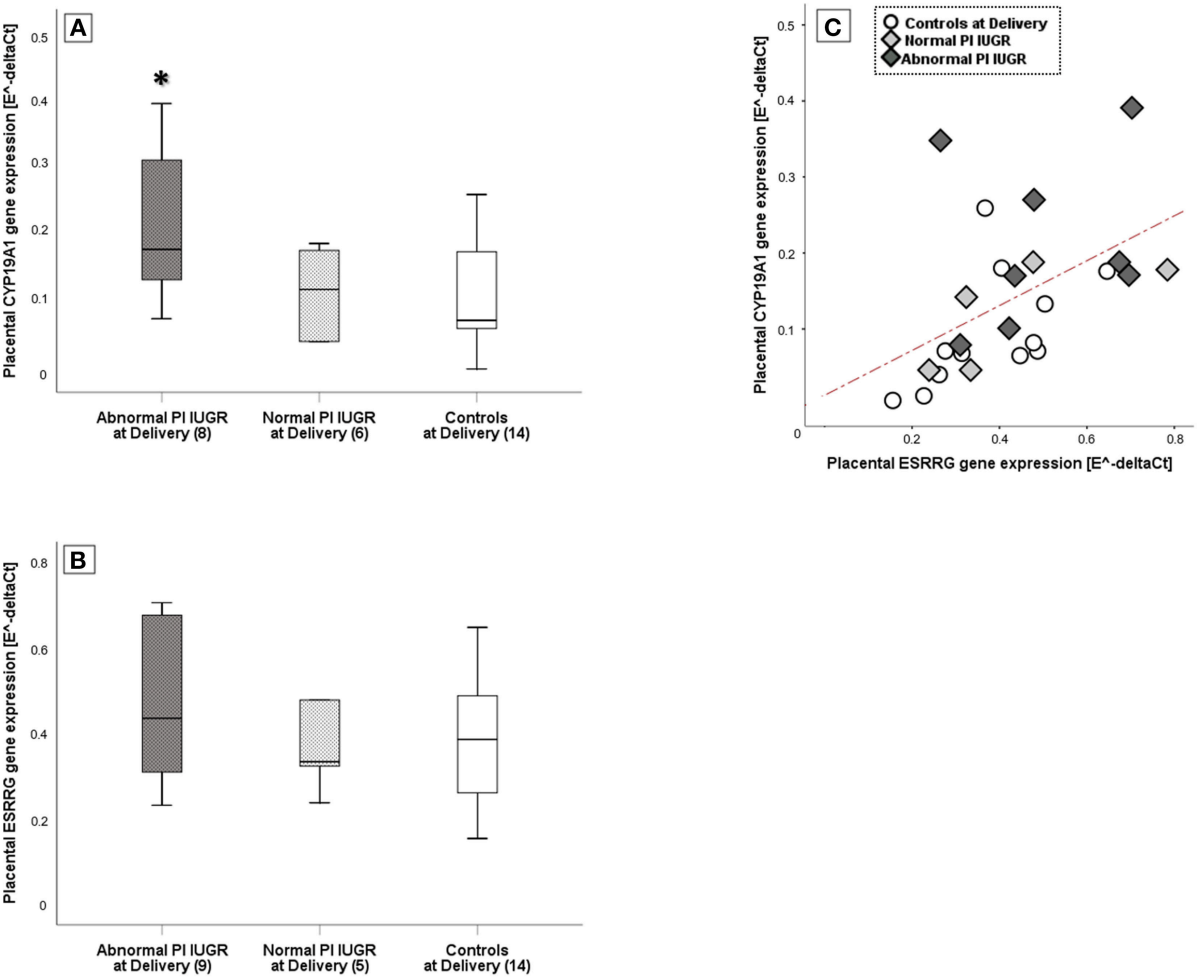
*ESRRG* and *CYP19A1* expression in placental tissue. **(A)** *CYP19A1* and **(B)** *ESRRG* mRNA levels in IUGR with Abnormal or Normal umbilical artery Pulsatility Index- PI and Controls at Delivery; relative mRNA levels derived according to geNorm method and data shown as Box Plots graphs. Statistical analysis by One-way between-groups ANOVA: ^*^p ≤ 0.05 vs. Controls at Delivery. **(C)** Significant positive correlation between *ESRRG* and *CYP19A1* expression in Controls at Delivery (○), Normal PI (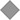), or Abnormal PI (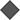) IUGR. Statistical analysis by Pearson product-moment correlation: *r* = 0.50/*p* ≤ 0.01.

A One-way ANOVA was performed to explore differences in *ESRRG* and *CYP19A1* tissue expressions among groups. There was a statistically significant difference among groups in *CYP19A1* levels [F_(2, 27)_ = 4.3, *p* = 0.02] with a large effect size (η^2^ = 0.50). Specifically, *post-hoc* test showed that *CYP19A1* levels were significantly increased in the most severe IUGR cases compared to both Normal PI IUGR and Controls (*p* = 0.02; Figure 2A) [Abnormal PI IUGR: 0.22 ± 0.11; Normal PI IUGR: 0.12 ± 0.06; Controls: 0.11 ± 0.08].

No significant differences were found for *ESRRG* gene expression [Normal PI IUGR: 0.43 ± 0.22; Abnormal PI IUGR: 0.47 ± 0.19; Controls: 0.38 ± 0.14] (Figure 2B). However, *ESRRG* placental levels significantly correlated to *CYP19A1* (*r* = +0.50/*p* = 0.01; Figure 2C).

#### Placental *CYP19A1* Expression in Relation to Fetal Sex

Two-way ANOVA showed a statistically significant interaction effect between growth restriction and fetal sex on placental *CYP19A1* [F_(2, 22)_ = 5.27, *p* = 0.01] with a large effect size (Partial η^2^ = 0.32). This suggests that the effect of IUGR on *CYP19A1* levels might also depend on fetal sex.

**Figure 3:**
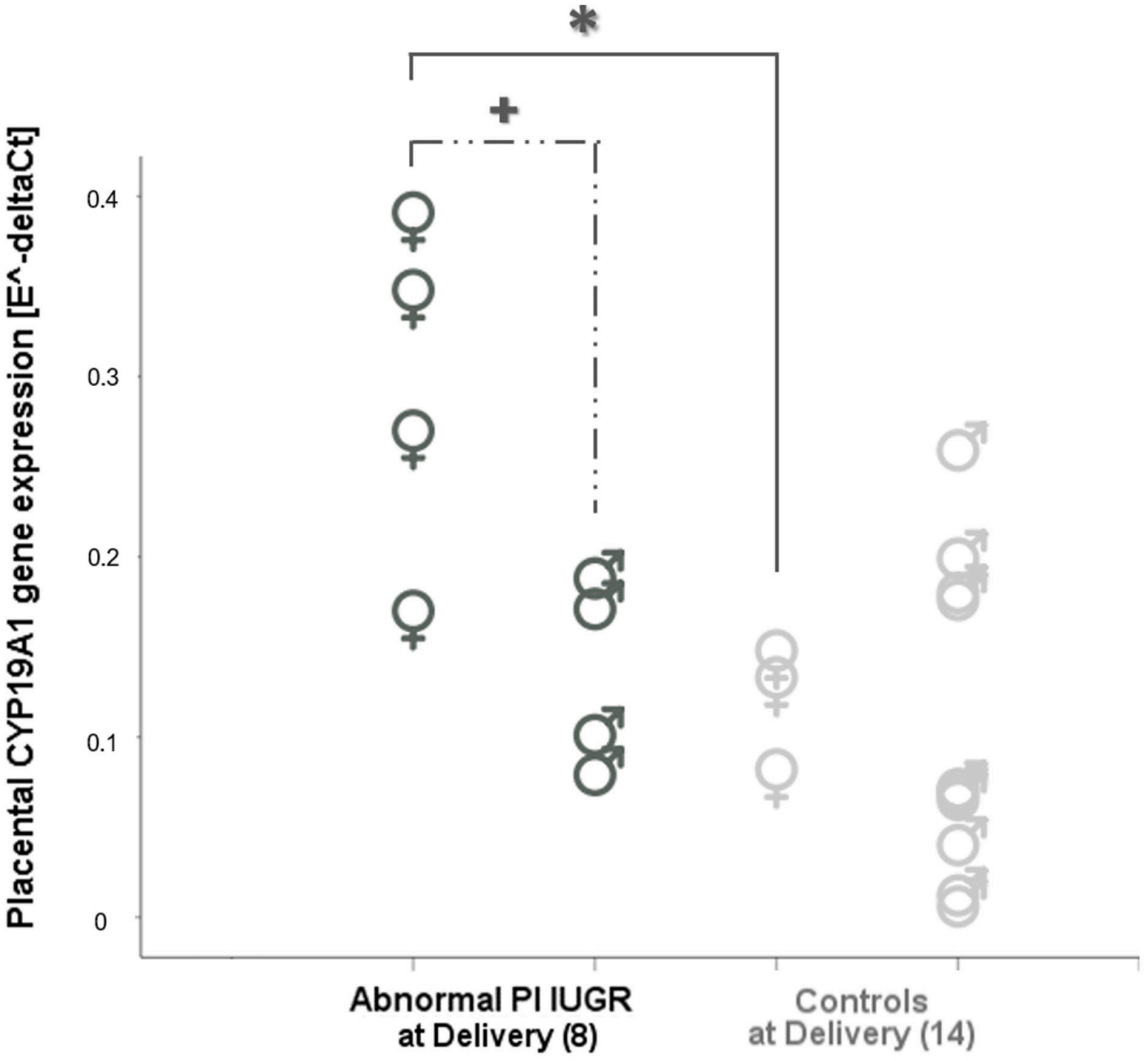
*CYP19* aromatase in placental tissue according to fetal sex. *CYP19A1* expression comparisons between female (**♀**) and male (**♂**) placentas in Abnormal PI IUGR and Controls at Delivery. Statistical analysis by Two-way between-groups ANOVA: *p < 0.05 Female Controls, or 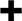 *p* < 0.05 Male IUGR vs. Female Abnormal PI IUGR.

This evidence was confirmed when comparing IUGR and Controls fetuses of the same sex: *CYP19A1* levels were significantly increased only in female fetuses when comparing Abnormal PI IUGR [0.30 ± 0.10] to Normal PI IUGR [0.08 ± 0.05] (*p* = 0.01, data not shown) and to Controls at Delivery [0.12 ± 0.04] (*p* = 0.03; Figure 3). Interestingly, *CYP19A1* gene expression was increased in female [0.30 ± 0.10] compared to male [0.14 ± 0.05] fetuses in the Abnormal PI IUGR subgroup (*p* = 0.03; Figure 3); no significant differences were found within the other subgroups.

## Discussion

In the present study we measured 17-Beta Estradiol levels in the maternal circulation, where it is mostly secreted after its placental production (46). We observed a significant decreased plasma concentration of E2 in IUGR pregnancies at third trimester. Moreover, we found a significant correlation between levels of 17-Beta Estradiol and placental efficiency, the ratio between fetal and placental weight, a parameter that has been associated with short- and long-term adverse outcomes (4, 47, 48). Since a growth-restricted placenta is known to present reduced efficiency (4), this might contribute to lower hormonal production, in a context of dysfunctional enzymatic metabolism. However, in our study 17-Beta Estradiol levels were not significantly different in IUGR maternal plasma compared to controls of similar gestational age. In the next future, we aim to enlarge this study population to verify the possible influence of gestational age on E2 levels.

However, previous studies have reported lower progesterone and estradiol concentrations in maternal plasma of growth-restricted pregnancies (25). Circulating estradiol levels have also been associated with the appropriate development of uterine artery diameters and blood flow at high-altitude (49), which are strictly related to placental development and consequently to fetal growth (50). Interestingly, 17-β Estradiol is also involved in nutrient supply. Indeed, in the mammary epithelium E2 activates the expression of *SNAT2* (Sodium-coupled Neutral amino acid Transporter 2) (51), an important transporter of neutral amino acids that is vital for a correct placental/fetal development. Altered *SNAT2* placental levels were previously reported by our group in IUGR placentas, its expression decreasing with growth-restriction severity (6). This may suggest a link between 17-β Estradiol and calorie restriction, a condition characterizing IUGR pregnancies, that needs further elucidation.

We then investigated the placental expression of *CYP19A1*, a gene involved in E2 production. *CYP19A1* expression is influenced by hypoxic conditions (34), while its activity is inhibited by ROS in placenta (52). Our study revealed significantly increased placental *CYP19A1* expression in IUGR pregnancies, which might represent a feedback signal aimed at supporting the disrupted estradiol production pathway. In a sheep model of hypoxia and oxidative stress, disturbances in ovarian aromatases function have been related to growth-restriction (24), supporting the hypothesis that alterations of the oxidative environment can cause changes in enzymes involved in steroidogenesis (53). Moreover, in a previous study investigating preeclamptic women, defective placental steroidogenesis has been proposed to explain altered steroidal plasma profiles, with accumulation of aromatase precursor-hormones (androstenedione and testosterone) opposite to diminished aromatase-metabolites (estrone and 17-β-Estradiol) (54). Our results support the hypothesis that *CYP19A1* placental changes can represent a compensatory response to the altered pathway of E2 synthesis in the IUGR placenta.

The Two-way ANOVA analysis revealed a significant combined effect of IUGR severity and fetal sex on *CYP19A1* levels. Fetal sex-related perturbations have been increasingly studied in preterm pregnancies with alternative findings (53, 55–57). Our results in IUGR placentas are consistent with changes noticed by Sathishkumar et al. in preeclamptic placentas (58). Interestingly, a sexual dimorphism has been reported in a maternal calorie-restriction rat model, where only male puppies showed a significant decrease of oxidative-stress plasma biomarkers (59). This might contribute to a different oxidative stress-related *CYP19A1* regulation in female and male placentas (60). Sex-specific steroids secretion or epigenetic sex-specific modulations might also represent mechanisms explaining our findings (61). Our preliminary findings of a placental sexual dimorphism involving sex steroids metabolism need deeper investigation in the next future.

In our study we also evaluated the Estrogen-Related Receptor “gamma” isoform, ESRRG, predominantly expressed in placenta (33) and indirectly linked to pregnancy estrogen pathways by supporting the expression of cytochrome P450 aromatase, one of the key enzymes for 17-Beta Estradiol placental production (62). *ESRRG* expression, inhibited by hypoxia, is also reported to be crucial in regulating trophoblast differentiation (63) and for placental vascularization and endocrine function (31, 34, 35). Although we found that *ESRRG* levels were significantly correlated to *CYP19A1*, we did not find significant differences in *ESRRG* levels among groups, therefore suggesting that further mechanisms might be involved in *CYP19A1* dysregulation in IUGR placentas.

This study was aimed at investigating placental changes of *ESRRG* and *CYP19A1* gene expressions in IUGR pregnancies, and their possible impact on maternal 17-Beta Estradiol plasma levels. Endocrine alterations likely can contribute to the growth-restricted phenotype. We therefore hypothesized alterations of placental steroidogenesis in IUGR compared to normal pregnancies. Our results partially support this hypothesis, showing that CYP19A1 placental changes could represent a compensatory response to the altered pathway of E2 synthesis in the IUGR placenta.

Data presented in this study, together with other recently reported evidences, represent a step forward for a deeper comprehension of placental steroidogenesis in IUGR. However, further molecular mechanisms could likely be involved, given the complexity of placental steroidogenesis and the multifactorial IUGR pathophysiology. The recent system biology approach, combining clinical, placental, and functional data simultaneously, will possibly gain insights into these complex pathways (64).

### Strengths and Limitations

A strength of this study are the strict inclusion and exclusion criteria that we applied for IUGR recruitment. Indeed, growth-restricted pregnancies were identified *in utero* through longitudinal measurements (abdominal circumferences <10^th^ percentile) and a shift from the growth curve >40 centiles. IUGR were further evaluated according to umbilical artery pulsatility index being classified in two groups of severity. This was a key point of this study, which allowed to avoid several biases due to the IUGR multifactorial features. However, the accurate selection of cases led to a limited sample size.

Gestational age represents a limit in the comparison between growth-restricted and control placentas. This is a limit occurring in all studies investigating human IUGR pregnancies, that cannot be avoided when studying placental samples. Therefore, results need to be evaluated taking into account this limitation.

## Conclusions

This study shows a relationship between 17-β Estradiol levels and placental efficiency in a population of intrauterine growth-restricted pregnancies. Changes in 17-β Estradiol are associated with a compensatory upregulation of *CYP19A1* or CYtochrome P450, an aromatase involved in E2 production. 17-β Estradiol and sex steroids are involved in pregnancy from implantation to parturition. Their biosynthesis and metabolism depend on complex pathways involving the fetus, the placenta and the mother. Additionally, we demonstrated a placental sexual dimorphism with effect on these pathways. Further investigation is needed to better understand the mechanisms behind the alterations we reported in the present preliminary study, in order to elucidate the pathophysiological strategies occurring in IUGR.

## Ethics Statement

This study was carried out in accordance with the recommendations of Regione Lombardia, Comitato Etico Locale ET/nb, with written informed consent from all subjects. All subjects gave written informed consent in accordance with the Declaration of Helsinki. The protocol named prot. no. 469/2010/52/AP was approved by the Comitato Etico Locale ET/nb.

## Author Contributions

GMA conceived, designed, and conducted the study by performing experiments, analyzing data, writing, and editing the manuscript. TL performed the electrochemiluminescence immunoassay and pre-analyzed the derived data. MIM and CP enrolled patients and collected biological samples. CN supported in planning the experiments and in editing the manuscript. FL contributed in performing samples preparation and data collection. TV supervised for the endocrinological data analysis. CM and IC were the project supervisors and provided a critical revision of the manuscript. All authors participated in the study and approved the final version of the manuscript declaring no conflicts of interest.

### Conflict of Interest Statement

The authors declare that the research was conducted in the absence of any commercial or financial relationships that could be construed as a potential conflict of interest.

## Abbreviations

CYP19A1: CYtochrome P450 aromatase gene
E2 or 17-β Estradiol: 17-Beta Estradiol
ER: Estrogen Receptor
ESRRG: Estrogen Related-Receptor Gamma gene
IUGR: Intrauterine Growth Restriction.
